# Craniofacial Effects of Zoledronic Acid on the Osteogenesis Imperfecta Mouse (−/−) Model of Severe Osteogenesis Imperfecta

**DOI:** 10.3390/biomedicines12122692

**Published:** 2024-11-25

**Authors:** Gaspard Jeannerod, Antoine Chretien, Grégoire André, Guillaume Mabilleau, Catherine Behets

**Affiliations:** 1 Pole of Morphology, Institute of Experimental and Clinical Research, UCLouvain, 1200 Brussels, Belgium; gaspard.jeannerod@gmail.com (G.J.); antoine.chretien@uclouvain.be (A.C.); gregoire.andre@uclouvain.be (G.A.); 2 Oniris, Inserm, UMR_S 1229–RMeS, REGOS, SFR ICAT, Univ Angers, Nantes Université, 49000 Angers, France; guillaume.mabilleau@unig-angers.fr; 3 Bone Pathology Unit, CHU Angers, 49000 Angers, France

**Keywords:** osteogenesis imperfecta, oim, zoledronic acid, craniofacial abnormalities

## Abstract

**Background:** Osteogenesis imperfecta (OI) is a rare genetic disorder affecting mainly type I collagen, which leads to bone fragility and deformities. OI patients also present craniofacial abnormalities such as macrocephaly and malocclusion. Recently, craniofacial dysmorphism was highlighted in the osteogenesis imperfecta mouse (oim), a validated model of the most severe form of OI. This study explores the impact of zoledronic acid (ZA), commonly administered to OI patients to increase bone mass and mechanical strength, on oim craniofacial structure. **Methods**: Fifteen oim received a single intravenous ZA injection (100 µg/kg) at 5 weeks (ZA group), while fifteen remained untreated (control). Before euthanasia at 14 weeks, in vivo computed tomography provided craniometric data. Post-euthanasia, heads underwent peripheral Quantitative Computed Tomography (pQCT); coronal decalcified sections through temporomandibular joints were analyzed (*n* = 6/mouse) after Masson’s trichrome staining (3 sections) or under polarized light to study collagen birefringence (3 sections). **Results**: In vivo craniometry highlighted the positive effect on vertical growth in ZA oim models as compared to untreated ones, with significant increases in mandibular length and incisor height and without any change in transversal dimensions. The pQCT scans showed the significantly higher total mineral density and cortical mineral density of the mandibular ramus in the ZA than the untreated group. Via microscopic analysis, the cranial vault was thicker and the collagen birefringence was higher in the ZA group than in the untreated group, but differences were not significant. **Conclusion**: To conclude, ZA had some beneficial effects on craniofacial vertical height and ramus density and, to a lower extent, on vault thickness, while transversal dimensions did not seem to be influenced by ZA intake. These data emphasize the need to consider the whole skeleton when treating OI patients.

## 1. Introduction

Osteogenesis imperfecta (OI) is a rare genetic disorder of connective tissue, caused by inherited mutations of the genes *COL1A1* or *COL1A2* in approximately 90% of cases. These latter encode the α1 and α2 chains of the type I collagen triple helix, the major structural protein in bone and dentin [1,2]. Recent studies indicate that mutations associated with osteogenesis imperfecta (OI) can affect various aspects of collagen, including its structure, posttranslational modification and folding, as well as bone mineralization and osteoblast differentiation [3]. These mutations result either in a defect of the collagen molecular structure or in a decrease in its production, leading to bone fragility, fracture, and deformation [4].

The severity of OI due to COL1A1 or COL1A2 mutations varies from mild cases that may go unnoticed to severe forms with perinatal death. The widely used clinical classification made by Sillence considers four groups: type I is the most common and non-deforming type; type II is associated with perinatal death; type III is a severe form leading to progressive skeletal deformity; and type IV is a moderate form with variable symptoms [2].

Specific craniofacial abnormalities have been identified in OI patients, such as macrocephaly, soft calvaria, the presence of Wormian bones, triangular facies, as well as malocclusion [2]. OI can also lead to abnormality of the craniofacial junction such as platybasia, basilar invagination, or basilar impression. They are considered secondary deformations resulting from bone softening [2,4,5].

**Figure 1:**
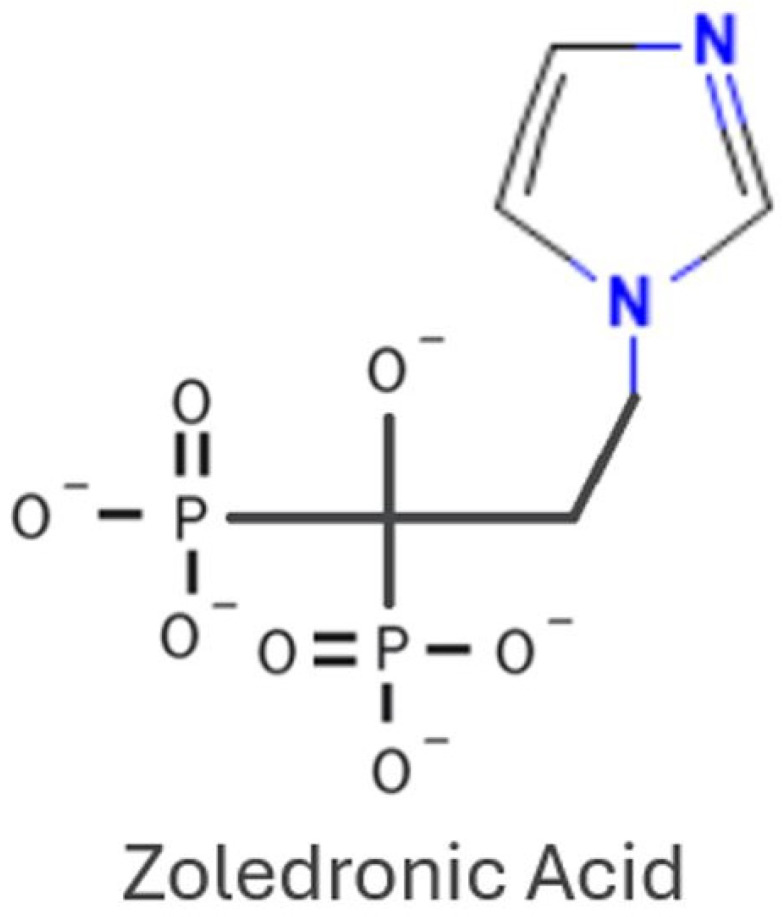
Chemical structure of zoledronic acid (C_5_H_10_N_2_O_7_P_2_·H_2_O; [1-hydroxy-2-(1H-imidazol-1-yl)ethane-1,1-diyl]bis(phosphonic acid) monohydrate).

Bisphosphonates (BPs) are synthetic analogues of pyrophosphate (Figure 1) with a high affinity for bone minerals. They belong to antiresorptive drugs and are widely prescribed to pediatric OI patients. There are two types of BPs: nitrogenous BPs (pamidronate, zoledronic acid or alendronate), disrupting osteoclast formation, survival, and cytoskeletal dynamic; and non-nitrogenous BPs (etidronate, clodronate), causing osteoclast apoptosis [6]. Once incorporated, they inhibit bone resorption and represent potent agents in managing diseases characterized by an accelerated bone turnover, such as osteoporosis and osteogenesis imperfecta [7]. Even though bisphosphonates do not interfere with collagen structure or production, they tend to significantly increase bone mass due to osteoclast inhibition [3]. While bone mass increases with bisphosphonate intake, certain studies do not support a statistically significant effect of BPs on fractures in OI [6]. The inhibition of osteoclasts slows down the remodelling of bone tissue, and, even though cortical width and trabecular number increase, it can result in bone brittleness as a long-term side effect [3]. The use of bisphosphonates is associated with several side effects, notably osteonecrosis of the jaw (ONJ). Although the incidence of ONJ is relatively high among oncology patients, it remains rare in those treated for osteoporosis, with an estimated occurrence ranging from 0.001% to 0.01%. [8]. To manage this risk, it is recommended to perform a dental evaluation and to resolve any dental issues before beginning bisphosphonate therapy. Patients should also be educated on maintaining oral hygiene and identifying early symptoms of ONJ [9]. Other side effects include an acute flu-like inflammatory response in 10–30% of patients after their first BP injection [7].

In bone tissue, the main functions of collagen proteins are to provide a scaffold allowing the formation of the mineral phase and to contribute to its mechanical properties [10]. During embryonic development, two different ossification processes occur: endochondral ossification, which develops on a pre-existing cartilaginous template, and intramembranous ossification, which directly converts a mesenchymal condensation into bone [11]. The flat bones of the cranial vault and face are formed by intramembranous ossification, while the long bones and bones of the skull base are formed by endochondral ossification. Although OI-related deformities of long bones have been well studied, their presence in bones of membranous origin is less known [12].

Recently, craniofacial dysmorphism was highlighted in osteogenesis imperfecta mouse models [13,14]. Among them, the oim one is considered a validated model of type III OI. In causing a shift in the last amino acids, the *COL1A2* gene mutation prevents the α2 chain from participating in the collagen triple helix. The homozygous oim only produces α1 homotrimers, which leads to abnormal bone matrix, low bone mass, skeletal deformities, short stature, and bone fragility. Moreover, the total mineral density and the total surface area of the parietal bone and mandibular ramus are significantly lower in oim than wild-type mice [14]. In addition to a significant diminution in size compared to the wild-type mice, oim are characterized by a reduction in cranium dimensions, including both horizontal and vertical craniofacial dimensions [14,15]. Consequently, the aim of the present study is to explore the potential beneficial effects of zoledronic acid intake on craniofacial features of female oim 9 weeks after treatment.

## 2. Materials and Methods

### 2.1. Animals and Procedures

This study was conducted with thirty homozygous female oim (strain B6C3Fe a/a-Col1a2oim/J). Males were excluded because a gender-related impact of treatment (antisclerostin antibody) was observed on bone in the oim model [16]. At 5 weeks of life, they were distributed into two groups (*n* = 15/group): the treated one received a single intravenous injection of zoledronic acid at 5 weeks (100 µg/kg, ZA group), and the control or OI group received no treatment. The animals were euthanized at 14 weeks, considering that they had reached their adult size. We did not investigate the effects on appendicular skeleton or the long-term consequences of treatment since the aim of the present work is to study the impact of ZA injection on craniofacial features during growth. The handling and housing of mice were undertaken in accordance with the Belgian federal law and protocols for the care of animals. All procedures were approved by the ethics committee for animal research of the Université Catholique de Louvain (2020/UCL/MD/02).

### 2.2. In Vivo Computed Tomography

All mice were scanned before euthanasia with an in vivo computed tomography (3D In Vivo Nano SPECT/CT^PLUS^, Mediso, Budapest, Hungary) tension = 65 kV) under sevoflurane inhalation anesthesia. Angular, linear, and area measurements were realized on coronal, transversal, and sagittal sections, as well as on a lateral view of the 3D reconstruction. All the anatomical landmarks and measurements are documented in Table 1. Some scans could not be used because of technical issues: several structures of interest were not scanned adequately due to animal positioning or movement during the acquisition.

### 2.3. Peripheral Quantitative Computed Tomography (pQCT)

After euthanasia, the head of each mouse was scanned using a pQCT Research SA+ scanner equipped with the XCT 5.4 built-in software (Stratec Medizintechnik GmbH, Pforzheim, Germany) device (XCT Research SA+, Stratec) at 65 kV and a nominal resolution of 70 µm. After the reconstruction of the tomogram, three coronal slices, spaced 150 µm apart, were acquired passing through the mandibular ramus, temporomandibular joint, and cranial vault. Three regions of interest (ROI) were selected and analyzed in each slice: cranial vault (CV), the right temporomandibular joint (TMJ) and the right mandibular ramus (MR). The threshold measurement was 570 mg/cm^3^ for cortical bone and 280 mg/cm^3^ for cancellous bone. The XCT 5.4 software allowed us to measure the total mineral density (including both cortical and trabecular bone), cortical mineral density (mg of hydroxyapatite/cm³), and cross-section area (mm^2^).

### 2.4. Histology

All heads were then decalcified (Osteo-RAL, RAL Diagnostics, Martillac, France) for three days and embedded in paraffin to perform coronal histological sections through the mandibular ramus, temporomandibular joint, and cranial vault. For each mouse, six 5 μm thick histological sections were selected: three of them were stained with Masson’s trichrome, and the other three were mounted unstained and observed under polarized light microscopy to study collagen organization through its birefringence. The birefringence is a property that allows us to study anisotropic structures and provides valuable information about tissue composition, orientation, and structural integrity. Appearing as brightness in the 8 bits image, the birefringence was quantified for each pixel with the grey values [0 (black)–255 (white)]. The cranial vault thickness was measured with the stained slices. All histological sections were imaged with the Axioplan microscope (ZEISS, Jena, Germany) with 1.25 mm objective lens and scanned using the Nikon Digital sight DS SMC camera (Nikon, Tokyo, Japan) with NIS-Element BR 3.0 software. The different measurements were performed with ImageJ software (version 1.53t).

### 2.5. Statistics

Statistical analyses were performed using an unpaired Student’s *t*-test or the non-parametric Mann–Whitney test. Linear correlation was calculated between cortical mineral density and mandibular height. Differences were considered significant if *p* < 0.05 (Prism 5.0, GraphPad Software, Inc., San Diego, CA, USA). In tables, data are presented as mean ± standard deviation (SD).

## 3. Results

### 3.1. In Vivo CT

Craniofacial height was increased after ZA administration, as compared to the control group, though not significantly. Indeed, both superior (+3.0%) and inferior (+4.2%) frontal heights were higher in ZA mice. No significant changes were noticed regarding craniofacial width (imad, icod, itd) (Table 2).

The area of the triangle between vertex and both articular tubercles was increased in ZA mice as compared to controls (+3.9%). However, data were at the limit of significance (*p* = 0.08). The cranial area was also slightly increased (+3.4%).

The sagittal skull area was significantly higher in ZA mice (+8.9%) than in control OI. However, the distance between the posterior point of the palate and that of the occipital bone did not vary significantly. Moreover, the position of the dens axis in relation to the basilar and occipital borders of the foramen magnum did not vary significantly between the two groups, but the data were slightly improved. There was no significant difference in the foramen magnum diameter between both groups.

The incisor height (Id-Li; +17.0%) and mandibular ramus length (Co-Gn; +10.45%) were significantly higher in the ZA group (*p* < 0.01) than in the OI control one. The mandibular height was slightly higher in the ZA group, but the difference was not significant (*p* = 0.07). The mandibular corpus length was not significantly modified by ZA intake. While the gonial angle was not significantly modified, the condylar angle appeared significantly decreased in the ZA group (*p* < 0.05).

### 3.2. pQCT

The administration of ZA did not induce any statistically significant effect on the cranial vault thickness, total mineral density, or cortical mineral density, as compared to the OI controls (Table 3).

In the ZA-treated group, significant higher values of total mineral density (+7.7%) and cortical mineral density (+4.5%) of the ramus were obtained as compared to the control group. Nevertheless, no statistically significant change was observed regarding the ramus bone width.

ZA-treated mice presented a higher total area (+8.3%) and total mineral density (+6.1%) of the temporomandibular joint than the controls. While data were at the limit of significance for total mineral density (*p* = 0.053), there was a significantly higher cortical mineral density of the temporomandibular joint in the ZA group (*p* = 0.03; +4.6%).

### 3.3. Correlation

**Figure 2:**
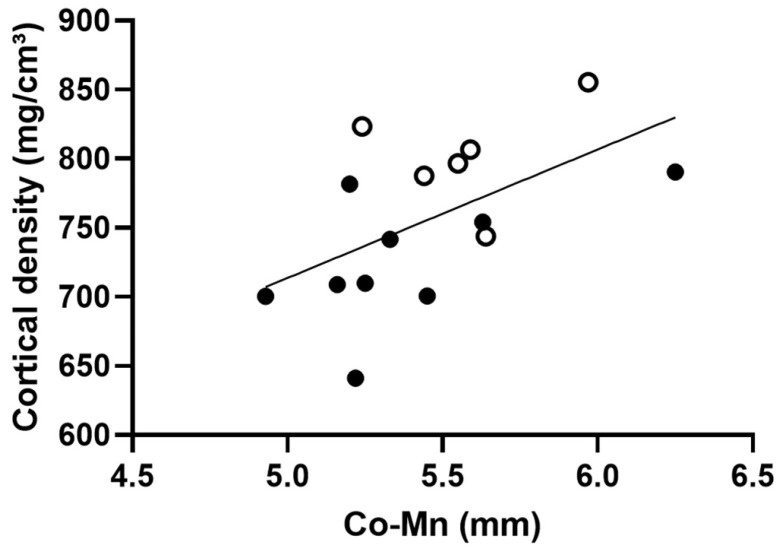
Correlation between the mandibular height (Co-Mn) and the cortical mineral density of mandibular ramus, R^2^ = 0.3, *p* < 0.05; full points are OI data while empty points are ZA ones.

Interestingly, there was a significant and positive correlation between the mandibular height and the cortical mineral density of mandibular ramus (Figure 2). Although the relation was weak, it suggests a link between the action of the treatment and the vertical growth.

### 3.4. Histomorphometry and Birefringence

**Figure 3:**
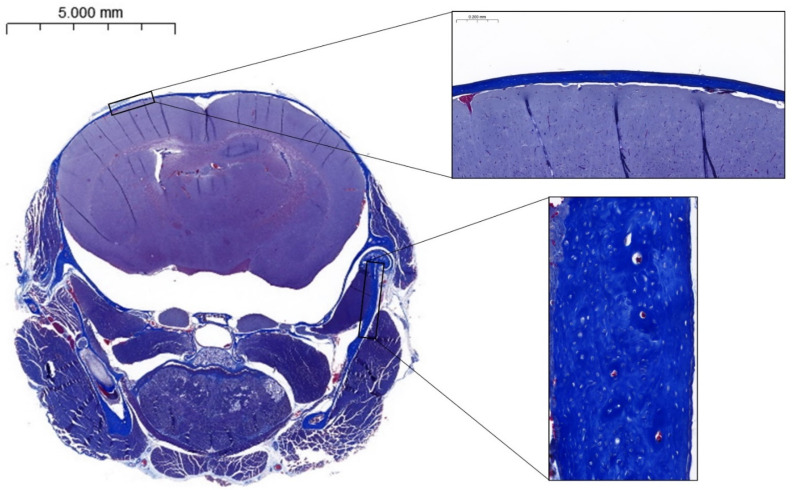
Coronal histological section of the calvaria of a mouse from the OI group stained with Masson’s trichrome, showing an absence of diploe in the calvaria and rounded osteocyte lacunae in the mandibular ramus.

The examination of the coronal sections revealed singular features of the calvaria and the ramus. The calvaria presented a single bone layer without diploe in both groups (Figure 3), and ZA intake seemed to induce an increase in calvaria thickness compared to controls (+26.1%). However, data were at the limit of significance (*p* = 0.08, Table 4).

Osteocyte lacunae presented a rounded shape in both groups. Furthermore, the bone matrix of the mandibular ramus did not exhibit a linear arrangement of collagen fibres, as visible with the anarchic disposition of osteocyte lacunae.

**Figure 4:**
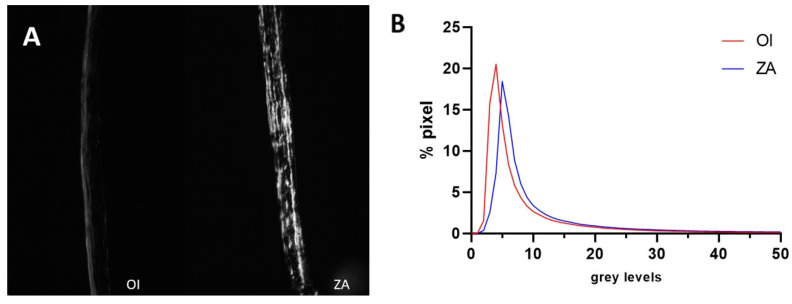
(**A**): coronal histological sections through the calvaria of OI and ZA mice, observed under polarized light; (**B**): Graph showing pixel distribution in the calvaria of OI and ZA groups, displaying grey levels of unstained sections observed under polarized light microscopy.

The birefringence of the calvaria was higher in ZA-treated mice than in the controls (+62.3%), while the difference was not statistically significant due to the important dispersion of data and low grey level values (Figure 4, Table 4). Consequently, the organization of collagen matrix in the calvaria was not quantitatively improved by the treatment.

## 4. Discussion

Craniometric scanning highlighted a significant positive effect of ZA on vertical growth in the treated group compared to the untreated group, showing increased mandibular length and incisor height. The midsagittal skull area was also significantly larger in the ZA group. The pQCT scans revealed a higher total mineral density and cortical mineral density in the mandibular ramus in the ZA group. Additionally, a significant positive correlation was found between mandibular height and cortical mineral density. Distinct histological characteristics were identified in both OI groups, along with a potential positive effect of the treatment on calvaria thickness compared to controls.

Some clinical studies have investigated craniofacial features associated with OI in humans. Waltimo-Siren et al. [12] and Chang [17] observed significant reductions in the anterior skull base height and overall vertical measurements, as well as a significant increase in the cranial base angle and a closing growth rotation in the mandibular region. They also observed that the intramembranous bones of the face are smaller in OI patients. While the correlation between prognathism (or class III malocclusion) and OI is contested by Waltimo-Siren’s study, conclusions vary widely. Chang et al. [17] observed a prognathism in 63% of cases in their study on craniofacial characteristics in OI patients.

In a mouse model of OI, Eimar et al. described an overall smaller head, a shortened anterior cranial base, a class III occlusion, and a mandibular side shift [15], which all are craniofacial features identified in OI patients. Morphometric analyses comparing craniofacial features of oim to wild-type mice validated oim as a representative model of human type III OI, in addition to the musculoskeletal features. Menegaz et al. described significantly smaller mandibular and skull lengths in oim compared to controls [18], validating the oim model for studying craniofacial characteristics in human OI. Future research could investigate foramen magnum stenosis in OI, as this is a serious complication of other skeletal dysplasias, such as achondroplasia [19]. Additionally, platybasia, basilar impression, and basilar invagination were shown to often be coexpressed in OI [20]. However, few studies investigated the effect of BPs on craniofacial features in OI [21]. The main hypothesis explaining the increased cranial base angle and reduced vertical measurement is the poor bone quality caused by the mutation, which leads to the progressive collapse of cranial structures [12]. ZA mice showed greater vertical dimensions than OI mice, suggesting that the effects of ZA injection on bone mineral density during growth may help compensate for poor bone quality, thus preventing cranial structures from collapse during growth, particularly the cranial base, which originates from endochondral ossification. Further research could focus on the effects of BPs on human craniofacial growth, particularly regarding vertical dimensions.

Oim teeth were previously shown to be smaller with shorter roots than those in wild-type mice, similar to findings in 50% of OI patients who show signs of dentinogenesis imperfecta [22,23]. Our study highlighted an increase in the mandibular incisor length in the ZA group, suggesting that ZA intake could impact tooth mineralization and structure. Since BPs are mainly administered during childhood in OI patients, this effect deserves attention in order to improve their tooth health.

Previous studies on adult wild-type mice have shown structural differences between cortical osteocytes in long bones and calvarial osteocytes. Calvarial osteocytes are typically round, while cortical osteocytes in long bones are more elongated [24] and their lacunae are mostly aligned with the principal loading axis [25]. This difference is thought to be due to the specific intramembranous ossification pathway in flat bones and differences in the loading patterns and osteocyte network activity in flat bones as opposed to long bones [24,26]. The spherical shape of calvarial osteocytes was also observed in both groups of this study, suggesting that the abnormal bone matrix in OI does not affect osteocyte morphology. A single table was observed in the calvaria of both OI and ZA groups, unlike previous observations on wild-type mice, where the usual external and internal tables are typically present in the calvaria.

The birefringence analysis of unstained sections showed no significant quantitative effect of ZA intake on collagen arrangement in the bone matrix, although there was an improved qualitative appearance. The effects of the treatment on osteocytes could also explain this positive trend of collagen matrix organization. BPs are administered in OI patients to increase bone quantity by inhibiting osteoclastic activity, thereby increasing the total mineral density [6]. Most studies on the effects of ZA have focused on long bones, but our study observed similar effects on intramembranous bones in a mouse model of OI.

pQCT results support the hypothesis that BP injections during growth positively affect the bone mineral density of craniofacial bones. Unlike the condylar and coronoid processes, which undergo endochondral ossification, the mandibular ramus primarily undergoes intramembranous ossification, similar to the bone tissue of the cranial vault [27,28]. Our findings suggest that BP intake also impacts osteoclastic metabolism in flat bones despite the morphological and structural differences in flat bone osteocytes as compared to long bone ones.

The craniofacial development is a complex and highly coordinated process controlled by genetic mechanism and environmental influence [29]. Concerning the cranial vault, the postnatal shape is mainly determined by extrinsic factors in response to extracranial muscular forces [30]. Taken together, the action of masticatory muscles, the growth of the tongue, and the facial soft tissue are determinants of mandible shape and size [29]. All these external factors influencing craniofacial growth could explain the beneficial effects of ZA administration observed concerning the vertical dimensions.

This study focused on ZA, which is intended to preserve and even increase bone volume and showed some beneficial effects on the craniofacial characteristics of oim. Unfortunately, some analysis highlighted differences which were at the limit of significance. This limitation may suggest either an insufficient number of data or measurement variability. Future research could investigate molecules that act on the collagen matrix quality and explore their effects on the craniofacial skeleton. New drugs with a potential impact on bone quality remain to be explored, as the current therapeutic options focus solely on increasing bone quantity.

## 5. Conclusions

This investigation provides valuable insights about the effects of ZA on the craniofacial skeleton in oim, emphasizing the potential of BP treatment to mitigate craniofacial skeletal abnormalities associated with OI. Despite the small number of mice, the observed benefits of ZA in enhancing bone mineral density and promoting vertical growth suggest that BP treatment may help to prevent the progressive collapse of craniofacial structures observed in OI. These findings encourage the further exploration of BP treatment for the management of craniofacial deformities in pediatric OI patients. Earlier intervention could improve facial growth patterns, functional outcomes, and consequently the overall quality of life. Future studies are still necessary to better understand the mechanisms underlying these effects, as well as to determine optimal dosing and timing to maximize therapeutic benefits without side effects.

## Figures and Tables

**Table 1:** Craniometric parameters assessed with CT scans, as defined by J. Fosséprez et al. [14].

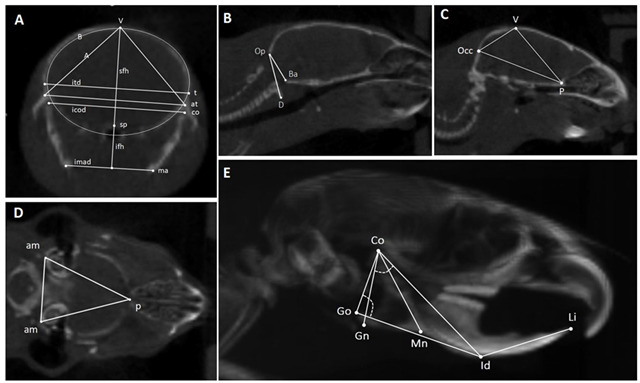				
	**Landmarks**	**Linear Measurements**	**Angle**	**Area**
A: Coronal slice through mandibular ramus, temporomandibular joints, and calvaria	t = temporo-parietal suture; sp = sphenoid bone; co = condylar process; ma = mandibular angle; at = articular tubercle.	sfh: superior frontal height; ifh: inferior frontal height; itd: intertemporal distance; icod: intercondylar distance; imad: intermandibular angles distance.	None	A = area of the triangle between V and both At; B is the frontal skull area.
B, C: Midsagittal slices	V = vertex; Occ = posterior point of the occipital bone; P = posterior point of the palatal bone; Op = occipital segment of the foramen magnum; Ba = basilar segment of the foramen magnum; D = anterior arch of atlas.	Occ-p = distance between the posterior point of the occipital bone and the posterior point of the palatal bone; Op-Ba = foramen magnum diameter.	D-Op-Ba angle = basilar angulation.	Sagittal skull area (Ssa) = area of the triangle between Occ, V, and P.
D: Transversal slice through both auditory meatuses	am = auditory meatus; p = posterior point of the palatal bone.	None	None	Transversal skull area (Tsa) = area of the triangle between Pp and both Am
E: 3D lateral view of the head	Co = condylar process; Go = the most posterior point of the gonial angle or of the angular process; Gn = the lowest point of the mandibular corpus; Mn = point in the deepest part of the antegonial notch curvature; Id = the most anterior point of the vestibular mandibular alveolar process; Li = upper incisor edge.	Co-Gn = mandibular ramus length; Co-Id = mandibular length; Go-Id = mandibular corpus length; Id-Li = extra-bone part of the lower incisor; Co-Mn = mandibular height.	Co-Go-Id = gonial angle; Gn-Co-Id = condylar angle.	None

**Table 2:** Cephalometric data assessed with CT scan.

(Distance in mm; Area in mm^2^; Angle in °)	OI	ZA	*p*
Coronal cut Superior frontal height (sfh) Inferior frontal height (ifh) Intertemporal distance (itd) Intercondylar distance (icod) Intermandibular angles distance (imad) Triangle vertex—both articular tubercules (A) Frontal skull area (B)	6.06 ± 0.23 3.86 ± 0.18 9.92 ± 0.21 9.60 ± 0.23 6.92 ± 0.36 23.34 ± 1.15 49.09 ± 2.47	6.24 ± 0.38 4.02 ± 0.34 9.88 ± 0.20 9.59 ± 0.24 6.94 ± 0.29 24.24 ± 1.50 49.41 ± 3.55	0.11 0.11 0.36 0.45 0.45 0.08 0.41
Midsagittal cut Sagittal skull area (Ssa) Sagittal length (Occ-P) Foramen magnum diameter (Op-Ba) Basilar angulation (D-Op-Ba, °)	26.06 ± 2.97 11.52 ± 0.38 3.70 ± 0.29 163.3 ± 27.7	28.37 ± 1.32 11.46 ± 0.41 3.74 ± 0.14 152.1 ± 29.1	<0.05 0.38 0.35 0.23
Transversal cut Transversal skull area (Tsa)	36.7 ± 1.8	37.6 ± 3.3	0.26
Lateral view of the head Extra-bone part of the lower incisor (Id-Li) Mandibular ramus length (Co-Gn) Mandibular length; (Co-Id) Mandibular corpus length (Go-Id) Mandibular height (Co-Mn) Gonial angle (Co-Go-Id, °) Condylar angle (Gn-Co-Id, °)	5.13 ± 0.47 4.40 ± 0.35 9.23 ± 0.67 8.59 ± 0.68 5.36 ± 0.37 87.7 ± 2.7 58.8 ± 3.9	5.97 ± 0.39 4.86 ± 0.31 9.38 ± 0.58 8.80 ± 0.49 5.61 ± 0.24 85.6 ± 3.6 55.6 ± 3.2	<0.01 <0.01 0.28 0.25 0.07 0.09 <0.05

Data are expressed as Mean ± SD, *n* = 10 for OI and *n* = 7 for ZA.

**Table 3:** Densitometric data assessed with pQCT.

	OI	ZA	*p*
Cross section area (mm^2^) ○ CV ○ MR ○ TMJ	3.03 ± 0.20 1.33 ± 0.29 1.54 ± 0.16	3.08 ± 0.39 1.37 ± 0.14 1.66 ± 0.23	0.71 0.64 0.11
Total mineral density (mg/cm³) ○ CV ○ MR ○ TMJ	525.0 ± 24.6 729.0 ± 41.6 692.6 ± 51.3	541.8 ± 52.2 785.5 ± 45.6 734.4 ± 55.9	0.29 <0.01 0.05
Cortical mineral density (mg/cm³) ○ CV ○ MR ○ TMJ	712.7 ± 15.8 931.1 ± 33.8 885.1 ± 51.9	730.3 ± 32.1 972.8 ± 39.7 926.5 ± 42.1	0.08 <0.01 0.03

Data are expressed as Mean ± SD, *n* = 14 for OI and *n* = 13 for ZA. CV: cranial vault; MR: mandibular ramus; TMJ: temporomandibular joint.

**Table 4:** Calvaria thickness and mean grey level measured in coronal sections observed either after staining or under polarized light, respectively.

	OI	ZA	*p*-Value
Calvaria thickness (mm)	0.61 ± 0.23	0.77 ± 0.22	0.08
Mean grey level (0–255)	10.69 ± 4.67	17.34 ± 12.52	0.20

Data are expressed as Mean ± SD; *n* = 15 for OI and *n* = 12 for ZA.

## Data Availability

Data are contained within the article.
